# Crystal structure and Hirshfeld surface analysis of 1-methyl-4-(2-methyl-10*H*-benzo[*b*]thieno[2,3-*e*][1,4]diazepin-4-yl)piperazin-1-ium 2,5-di­hydroxy­benzoate propan-2-ol monosolvate

**DOI:** 10.1107/S205698902000818X

**Published:** 2020-06-30

**Authors:** V. Natchimuthu, N. Sharmila, S. Ravi

**Affiliations:** a Department of Physics, M.Kumarasamy College of Engineering, Karur 639113, Tamil Nadu, India; b Department of Physics, Shrimati Indira Gandhi College, Tiruchirappalli 620 002, Tamilnadu, India; c Postgraduate and Research Department of Physics, National College (Autonomous), Tiruchirappalli 620 001, Tamilnadu, India

**Keywords:** crystal structure, olanzapine, salt, hydrogen bond, Hirshfeld surface

## Abstract

The asymmetric unit of the title salt, C_17_H_21_N_4_S^+^·C_7_H_5_O_4_^−^·C_3_H_7_OH, consists of an olanzapinium cation, an independent 2,5-di­hydroxy­benzoate anion and a solvent isopropyl alcohol mol­ecule. The central seven-membered heterocycle is in a boat conformation, while the piperazine ring displays a distorted chair conformation. The dihedral angle between the benzene and thiene rings flanking the diazepine ring is 52.58 (19)°. In the crystal, the anions and cations are connected by N—H⋯O and O—H⋯O hydrogen bonds, forming a three-dimensional network.

## Abstract 2

The asymmetric unit of the title salt consists of an olanzapinium cation, an independent 2,5 di­hydroxy­benzoate anion and a solvent isopropyl alcohol mol­ecule. The central seven-membered heterocycle is in a boat conformation, while the piperazine ring displays a distorted chair conformation. The dihedral angle between the benzene and thiene rings flanking the diazepine ring is 52.58 (19)°. In the crystal, the anions and cations are connected by N—H⋯O and O—H⋯O hydrogen bonds, forming a three-dimensional network.

## Chemical context   

Olanzapine is an atypical anti­psychotic with indications for the treatment of schizophrenia, acute mania and the prevention of relapse in bipolar disorder. Olanzapine is structurally similar to clozapine, but is classified as a thienobenzodiazepine. Reviews on olanzapine in the management of bipolar disorders (Narasimhan *et al.*, 2007 ▸) and olanzapine-associated toxicity and fatality in overdose (Chue & Singer, 2003 ▸) have been published. Olanzapine, the pharmaceutically active component of the title compound, a thienobenzodiazepine derivative, along with clozapine, quetiapine, risperidone and ziprasidone, belongs to the newer generation of atypical anti­psychotic agents (Chakrabarti *et al.*, 1980 ▸; Callaghan *et al.*, 1999 ▸; Kennedy *et al.*, 2001 ▸; Tandon & Jibson, 2003 ▸).

These atypical anti­psychotic agents, in comparison with the older generation, show greater efficacy against both positive and negative symptoms of schizophrenia (a debilitating mental disorder) as well as associated cognitive deficits and are virtually devoid of extrapyramidal symptoms (Tandon, 2002 ▸). The therapeutic action of olanzapine against the symptoms of schizophrenia is thought to be due to its high affinity for dopamine­rgic D2 and serotonergic 5-HT2A receptor systems implicated in the pathogenesis of this disease (Bever & Perry, 1998 ▸).

The crystal structures of 2-methyl-4-(4-methyl­piperazin-1-yl)-10*H*-thieno[2,3-*b*][1,5]benzodiazepine methanol solvate monohydrate (Capuano *et al.*, 2003 ▸), polymorphic form II of 2-methyl-4-(4-methyl-1-piperazin­yl)-10*H*-thieno[2,3-*b*][1,5]benzodiazepine (Wawrzycka-Gorczyca *et al.*, 2004*a* ▸), 2-meth­yl-4-(4-methyl-1-piperazin­yl)-10*H*-thieno[2,3-*b*][1,5] benzo­di­azepine methanol solvate (Wawrzycka-Gorczyca *et al.*, 2004*b* ▸), olazipinium nicotinate (Ravikumar *et al.*, 2005 ▸), olanzapine and its solvates (Wawrzycka-Gorczyca *et al.*, 2007 ▸), highly soluble olanzapinium maleate crystalline salts (Thakuria & Nangia, 2011*a* ▸) and polymorphic form IV of olanzapine (Thakuria & Nangia, 2011*b* ▸) have been reported. In view of the importance of olanzapine, this paper reports the crystal structure of the title salt, C_17_H_21_N_4_S^+^·C_7_H_5_O_4_^−^·C_3_H_7_OH, (I) 
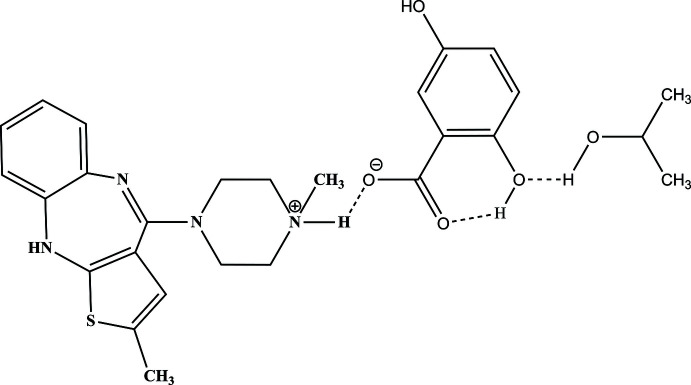
.

## Structural commentary   

**Figure 1:**
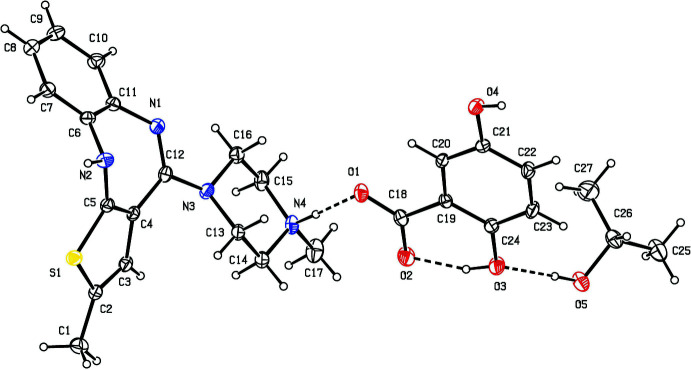
The mol­ecular structure of (I), with displacement ellipsoids for the non-H atoms drawn at the 30% probability level. Hydrogen bonds (Table 1 ▸) are shown as dashed lines.

A perspective view of (I), with the atomic numbering scheme, is illustrated in Fig. 1 ▸. The asymmetric unit comprises an olanzapinium cation, an independent 2,5-di­hydroxy­benzoate anion and a solvent isopropyl alcohol mol­ecule. The central seven-membered (N1/C11/C6/N2/C5/C4/C12) heterocycle is in a boat conformation with puckering parameter *Q* = 0.715 (3) Å while the six-membered piperazine ring, N3/C13/C14/N4/C15/C16, adopts a distorted chair conformation with puckering parameters *Q* = 0.564 (3) Å, θ = 175.3 (3)°, φ = 200 (4)°*.* The dihedral angle between the benzene and thiene rings flanking the diazepine ring is 52.58 (19)°. This is similar to the values observed in the related structure olanzapinium dipicrate (II) [58.7 (9)°] *.* The dihedral angles between the plane of the four C atoms in the piperazine ring and the planes of the benzene and thio­phene rings are 27.04 (13) and 33.36 (18)°, respectively. In the 2,5-di­hydroxy­benzoate, the mean plane of the C18–O1–O2 group is twisted by 4.7 (5)° from that of the benzene ring (C19–C24). The bond lengths and bond angles of the thiene and piperazine rings of compound (I) are also comparable with the values observed for related structures (Kavitha *et al.*, 2013 ▸; Ravikumar *et al.*, 2005 ▸).

**Figure 2:**
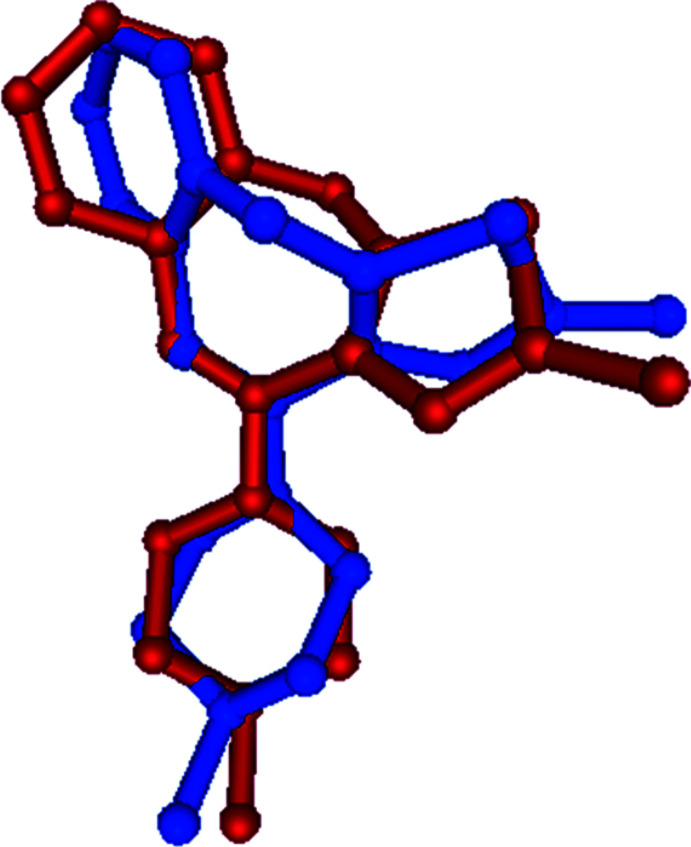
A superimposed fit of (I) (red) and the related structure (II) (blue).

**Figure 3:**
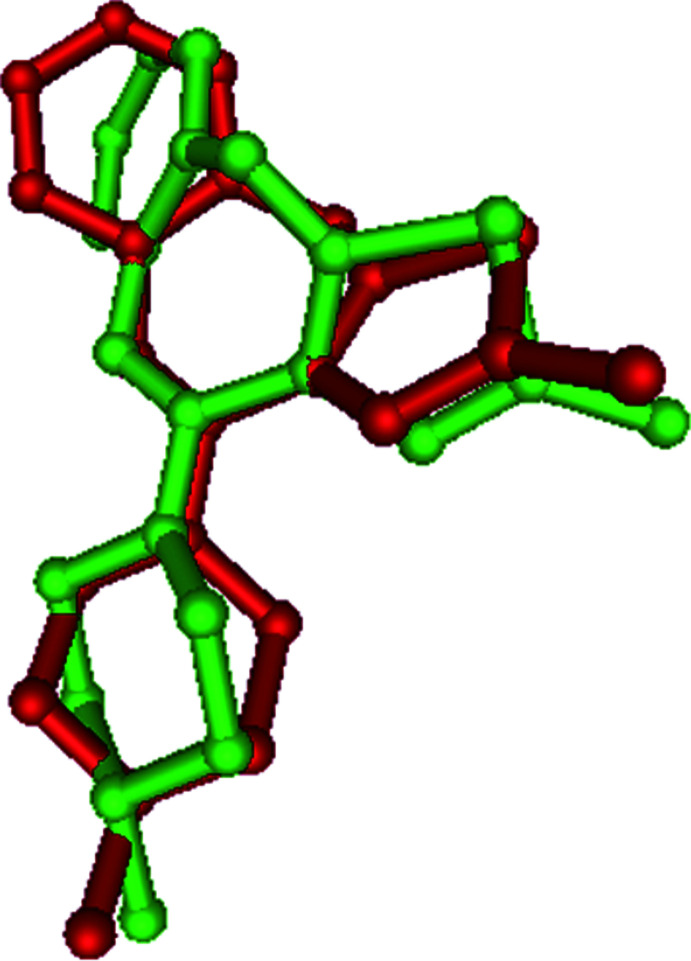
A superimposed fit of (I) (red) and the related structure (III) (green).

The superimposed fit (Gans & Shalloway, 2001 ▸) of the olaza­pine group of (I) (atoms C1–C8, N1, O1 and O2) gives an r.m.s deviation of 1.179 Å with olanzapinium dipicrate (II) (Kavitha *et al.*, 2013 ▸) (Fig. 2 ▸) and 1.175 Å with olazipinium nicotinate (III) (Ravikumar *et al.*, 2005 ▸) (Fig. 3 ▸). The larger r.m.s deviation with the related structure may be due to the different substitution of groups on the olanzapinium cation.

## Supra­molecular features   

**Figure 4:**
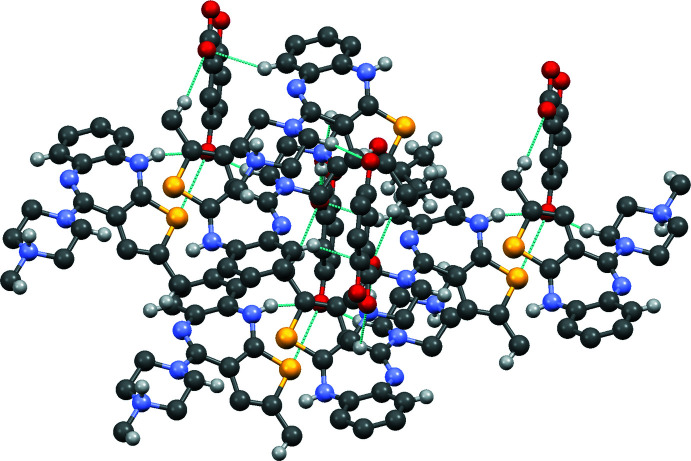
Crystal packing of (I), showing the C—H⋯O hydrogen bonds [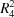 (22) ring motif, 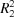 (15) and 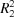 (7) ring motifs; Table 1 ▸] as dashed lines. H atoms not involved in these inter­actions have been omitted for clarity.

**Figure 5:**
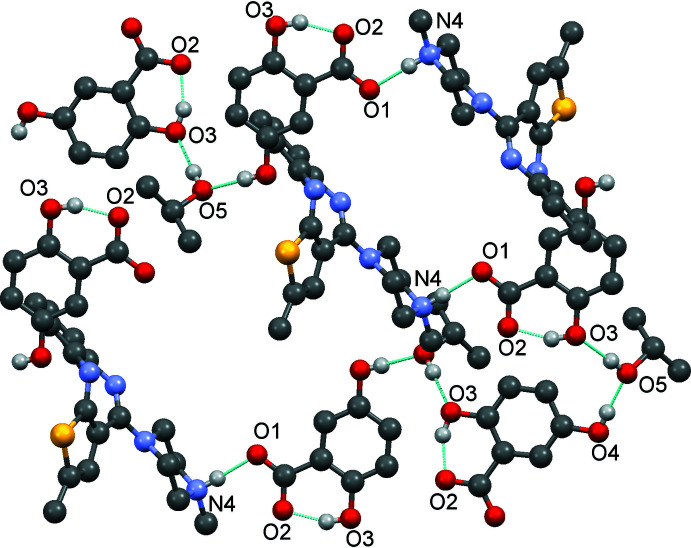
Crystal packing of (I), showing the O—H⋯O and N—H⋯O hydrogen bonds (Table 1 ▸) as dashed lines; the shortest contacts between O2 and O3 give rise to an *R*(6) motif. H atoms not involved in these inter­actions have been omitted for clarity.

In the crystal, the anions and cations are connected by C—H⋯O, N—H⋯O and O—H⋯O hydrogen bonds (Table 1 ▸), forming a three-dimensional network. The inter­action between C1—O1 and C10—O1 *via* atoms H1*A* and H10 encloses an 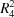 (22) ring motif. In addition, the inter­action between C1—O1 and C13—O4 *via* atoms H1*A* and H13*A* forms an 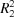 (15) ring motif and that between C17—O2 and N4—O1 *via* atoms H17*B* and H4*N* encloses an 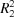 (7) ring motif (Fig. 4 ▸). The atoms O2 and O3, O4, O5 and O3, O5 are connected through H3*A*, H4*A* and H5, forming an inter­molecular ring motif. The contact between atoms N4 and O1 *via* H4*N* generates parallel chains to form a three dimensional network (Fig. 5 ▸).

## Database survey   

A search of the Cambridge Crystallographic Database (CSD version 5.41, last update March 2020; Groom *et al.*, 2016 ▸) gave only twenty-two entries based on the olanzapine drug mol­ecule. They include salts with gallic acid: {CSD refcodes SUKPEW, 1-methyl-4-(2-methyl-10*H*-thieno[2,3-*b*][1,5]ben­zo­diazepin-4-yl)piperazin-1-ium 2,4,6-tri­hydroxy­benzoate, and SUKPOG, 1-methyl-4-(2-methyl-10*H*-thieno[2,3-*b*][1,5]benzodiazepin-4-yl)piperazin-1-ium 3,4,5-tri­hydroxy­benzoate dihydrate; Sarmah *et al.*, 2020 ▸}, with mono and dihy­droxy benzoic acid {FABJUQ, 1-methyl-4-(2-methyl-10*H*-thieno[2,3-*b*][1,5]benzodiazepin-4-yl) piperazin-1-ium 4-hy­droxy­benzoate aceto­nitrile solvate, FABJIE, 1-methyl-4-(2-methyl-10*H*-thieno[2,3-*b*][1,5]benzodiazepin-4-yl) piperazin-1-ium 2,5di­hydroxy­benzoate, FABJEA, 1-methyl-4-(2-methyl-10*H*-thieno[2,3-*b*][1,5]benzodiazepin-4-yl)piperazin-1-ium 2,4-di­hydroxy­benzoate and FABJOK, 1-methyl-4-(2-methyl-10*H*-thieno[2,3-*b*][1,5]benzodiazepin-4-yl) piperazin-1-ium 2,6di­hydroxy­benzoate; Sarmah *et al.*, 2016 ▸}, with nicotinic acid {TAQNUV, 1-methyl-4-(2-methyl-10*H*-thieno[2,3-*b*][1,5]ben­zo­diazepin-4-yl) hexa­hydro­pyrazin-1-ium nicotinate; Ravikumar *et al.*, 2005 ▸}, with pyrazinoic acid (SUKPAS; Sarmah *et al.*, 2020 ▸) and with other carb­oxy­lic acids (AMIYUR and AMIZAY; Thakuria *et al.*, 2011*a* ▸ and Sarmah *et al.*, 2020 ▸; FABKAX and FABKEB; Sarmah *et al.*, 2016 ▸ *;* FHIRYUE, HIRZAL, HIRZEP and HIRZIT; Thakuria *et al.*, 2013 ▸; JIXROY; Sridhar & Ravikumar, 2007 ▸; LESQIL; Kavitha *et al.*, 2013 ▸; PEWPUF, PEWQAM and PEWQEQ; Sarmah *et al.*, 2018 ▸; TAQNUV; Ravikumar *et al.*, 2005 ▸ *).* Among them, the crystal structures of PEWQEQ, PEWQAM, HIRZIT, FABJUQ, SUKPIA, SUKPOG, FABKEB, HIRZEP and PEWQAM contain solvent mol­ecules.

## Hirshfeld surface (HS) analysis   

**Figure 6:**
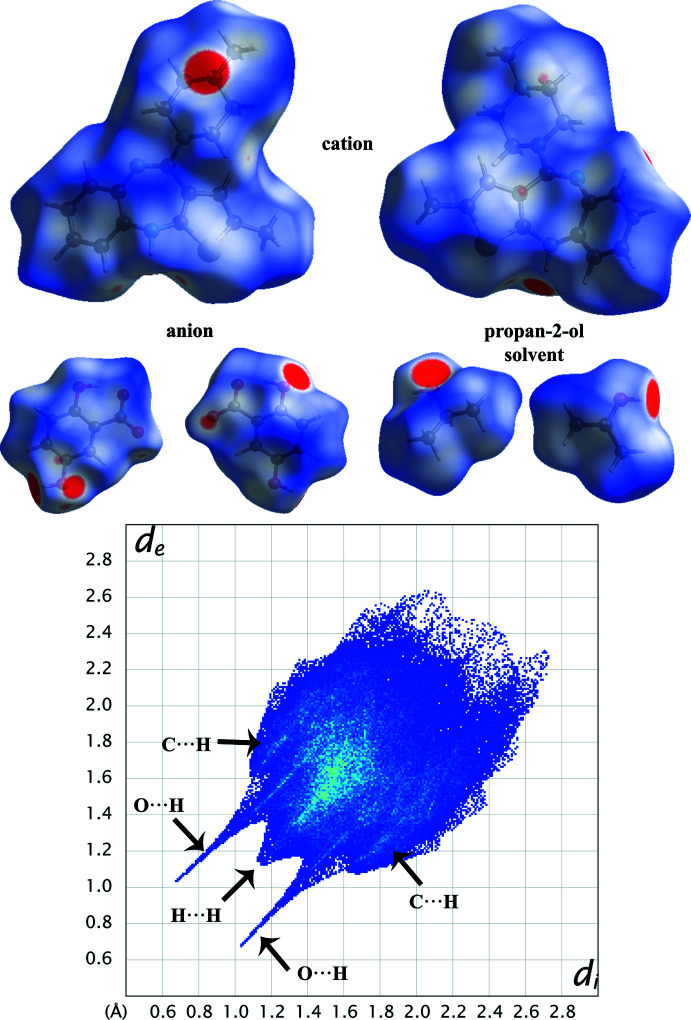
Views of the Hirshfeld surfaces of title compound (I) mapped with *d*_norm_ in two different orientations. The HS is plotted in the range −0.1500 to 1.4938 a.u.

**Figure 7:**
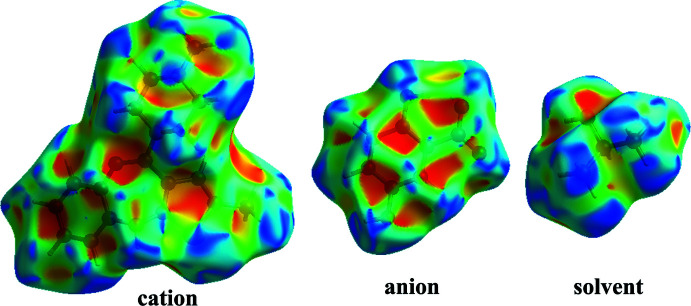
Views of the shape-index diagram of title compound (I).

The HS analysis (McKinnon *et al.*, 1998 ▸, 2004 ▸, 2007 ▸; Spackman & Jayatilaka, 2009 ▸) was performed to understand the inter­molecular inter­actions in the crystal structure of (I) and was constructed in the crystal environment using *CrystalExplorer 17.5* (Turner *et al.*, 2017 ▸). The various non-covalent inter­actions are qu­anti­fied with decomposed, two-dimensional fingerprint plots (Spackman & McKinnon, 2002 ▸). The HS plotted over *d*_norm_ is shown in Fig. 6 ▸ with red areas indicating distances shorter (in closer contact) and blue those longer (distant contact) than the van der Waals radii. The contacts with distances equal to the sum of van der Waals radii are indicated in white (Venkatesan *et al.*, 2016 ▸). From Fig. 6 ▸, the bright-red spots appearing near the hydrogen atoms H2*N*, H4*N*, H10, and H13 in the cation indicate that these hydrogen atoms are involved in the inter­molecular inter­actions. The shape-index (SI) diagram, a tool to visualize π–π stacking inter­actions, for the cation, anion and solvent mol­ecule is shown in Fig. 7 ▸. No adjacent red and blue triangles are seen, indicating that no π–π inter­actions are present, which is in agreement with the experimental findings. The overall two-dimensional fingerprint (2D–FP) plots are illustrated in Fig. 6 ▸. The H⋯H contacts make the highest contribution (53.8%) to the total crystal packing (broad peaks at *d*_e_+ *d*_i_ = ∼2.3 Å). The second highest contribution is from H⋯C/C⋯H contacts (21.8%) and is indicated by the broad wing-like structure at *d*_e_+ *d*_i_ = ∼2.6 Å. The symmetrical sharp spikes at *d*_e_+ *d*_i_ = ∼1.6 Å are attributed to H⋯O/O⋯H contacts (14.3%).

## Synthesis and crystallization   

Olanzapine (156 mg, 0.5 mmol) and 2,5-di­hydroxy­benzoic acid (77 mg, 0.5 mmol) were dissolved in 20 mL of isopropyl alcohol and stirred magnetically for 5 h at 330 K. The mixture was kept aside for two days at room temperature and the salt formed was filtered off and dried. The compound was recrystallized from (1:1) isopropyl alcohol/DMF by slow evaporation at room temperature (m.p. 373–375 K).

## Refinement   

Crystal data, data collection and structure refinement details are summarized in Table 2 ▸. The N-bound and O-bound H atoms were located in a difference-Fourier map and freely refined. The C-bound H atoms were included in calculated positions and treated as riding atoms: C—H = 0.93–0.97 Å with *U*_iso_(H) = 1.5*U*_eq_(C) for methyl H atoms and = 1.2*U*_eq_(C) for other H atoms.

## Supplementary Material

Crystal structure: contains datablock(s) I. DOI: 10.1107/S205698902000818X/mw2162sup1.cif

Structure factors: contains datablock(s) I. DOI: 10.1107/S205698902000818X/mw2162Isup2.hkl

CCDC reference: 2010899

Additional supporting information:  crystallographic information; 3D view; checkCIF report

## Figures and Tables

**Table 1:** Hydrogen-bond geometry (Å, °)

*D*—H⋯*A*	*D*—H	H⋯*A*	*D*⋯*A*	*D*—H⋯*A*
C1—H1*A*⋯O1^i^	0.96	2.64	3.579 (4)	168
C10—H10⋯O1^ii^	0.93	2.52	3.316 (4)	143
C13—H13*A*⋯O4^i^	0.97	2.62	3.233 (4)	122
C17—H17*B*⋯O2	0.96	2.63	3.216 (4)	120
N2—H2⋯O4^iii^	0.87 (3)	2.28 (3)	3.088 (4)	156 (3)
N4—H4⋯O1	0.89 (4)	1.77 (4)	2.660 (3)	178 (4)
O3—H3*A*⋯O2	0.89 (4)	1.63 (4)	2.479 (3)	156 (4)
O4—H4*A*⋯O5^iv^	0.85 (4)	1.84 (4)	2.682 (3)	173 (4)
O5—H5⋯O3^v^	0.88 (4)	1.88 (4)	2.764 (3)	178 (3)

Symmetry codes: (i) 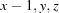; (ii) 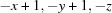; (iii) 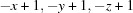; (iv) 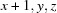; (v) 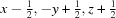.

**Table 2:** Experimental details

Crystal data	
Chemical formula	C_17_H_21_N_4_S^+^·C_7_H_5_O_4_^−^·C_3_H_8_O
*M*_r_	526.64
Crystal system, space group	Monoclinic, *P*2_1_/*n*
Temperature (K)	294
*a*, *b*, *c* (Å)	8.4867 (6), 29.764 (2), 10.6334 (8)
β (°)	94.381 (1)
*V* (Å^3^)	2678.1 (3)
*Z*	4
Radiation type	Mo *K*α
μ (mm^−1^)	0.17
Crystal size (mm)	0.15 × 0.14 × 0.06

Data collection	
Diffractometer	Bruker *SMART* CCD area-detector diffractometer
Absorption correction	Multi-scan (*SADABS*; Bruker, 2008 ▸)
*T*_min_, *T*_max_	0.96, 0.98
No. of measured, independent and observed [*I* > 2σ(*I*)] reflections	25175, 4560, 4081
*R*_int_	0.040
(sin θ/λ)_max_ (Å^−1^)	0.588

Refinement	
*R*[*F* ^2^ > 2σ(*F* ^2^)], *wR*(*F* ^2^), *S*	0.069, 0.143, 1.31
No. of reflections	4560
No. of parameters	358
H-atom treatment	H atoms treated by a mixture of independent and constrained refinement
Δρ_max_, Δρ_min_ (e Å^−3^)	0.32, −0.21

Computer programs: *SMART* (Bruker, 2008 ▸), *SAINT* (Bruker, 2008 ▸), *SHELXS97* (Sheldrick, 2008 ▸), *SHELXL2018/3* (Sheldrick, 2015 ▸), *QMOL* (Gans & Shalloway, 2001 ▸), *Mercury* (Macrae *et al.*, 2020 ▸), *ORTEPIII* (Burnett & Johnson, 1996 ▸), *WinGX* publication routines (Farrugia, 2012 ▸) and *PLATON* (Spek, 2020 ▸).

## supplementary crystallographic information

### Crystal data

**Table:**

C_17_H_21_N_4_S^+^·C_7_H_5_O_4_^−^·C_3_H_8_O	*F*(000) = 1120
*M**_r_* = 526.64	*D*_x_ = 1.306 Mg m^−^^3^
Monoclinic, *P*2_1_/*n*	Mo *K*α radiation, λ = 0.71073 Å
*a* = 8.4867 (6) Å	Cell parameters from 4689 reflections
*b* = 29.764 (2) Å	θ = 2.3–24.2°
*c* = 10.6334 (8) Å	µ = 0.17 mm^−^^1^
β = 94.381 (1)°	*T* = 294 K
*V* = 2678.1 (3) Å^3^	Solid, white
*Z* = 4	0.15 × 0.14 × 0.06 mm

### Data collection

**Table:**

Bruker SMART CCD area-detector diffractometer	4081 reflections with *I* > 2σ(*I*)
Radiation source: fine focus sealed tube	*R*_int_ = 0.040
ω and φ scan	θ_max_ = 24.7°, θ_min_ = 2.0°
Absorption correction: multi-scan (SADABS; Bruker, 2008)	*h* = −9→9
*T*_min_ = 0.96, *T*_max_ = 0.98	*k* = −35→35
25175 measured reflections	*l* = −12→12
4560 independent reflections	

### Refinement

**Table:**

Refinement on *F*^2^	Primary atom site location: structure-invariant direct methods
Least-squares matrix: full	Secondary atom site location: difference Fourier map
*R*[*F*^2^ > 2σ(*F*^2^)] = 0.069	Hydrogen site location: mixed
*wR*(*F*^2^) = 0.143	H atoms treated by a mixture of independent and constrained refinement
*S* = 1.31	*w* = 1/[σ^2^(*F*_o_^2^) + (0.0423*P*)^2^ + 1.7858*P*] where *P* = (*F*_o_^2^ + 2*F*_c_^2^)/3
4560 reflections	(Δ/σ)_max_ = 0.001
358 parameters	Δρ_max_ = 0.32 e Å^−^^3^
0 restraints	Δρ_min_ = −0.21 e Å^−^^3^

### Special details

**Table:**

Geometry. All esds (except the esd in the dihedral angle between two l.s. planes) are estimated using the full covariance matrix. The cell esds are taken into account individually in the estimation of esds in distances, angles and torsion angles; correlations between esds in cell parameters are only used when they are defined by crystal symmetry. An approximate (isotropic) treatment of cell esds is used for estimating esds involving l.s. planes.

### Fractional atomic coordinates and isotropic or equivalent isotropic displacement parameters (Å^2^)

**Table:**

	*x*	*y*	*z*	*U*_iso_*/*U*_eq_	
C1	−0.3762 (4)	0.47317 (12)	0.3380 (4)	0.0585 (9)	
H1A	−0.390099	0.447460	0.283753	0.088*	
H1B	−0.459922	0.494268	0.317993	0.088*	
H1C	−0.378228	0.463851	0.424267	0.088*	
C2	−0.2208 (3)	0.49488 (10)	0.3192 (3)	0.0406 (7)	
C3	−0.1143 (3)	0.48603 (10)	0.2353 (3)	0.0385 (7)	
H3	−0.128823	0.463584	0.174715	0.046*	
C4	0.0236 (3)	0.51399 (9)	0.2470 (3)	0.0352 (7)	
C5	0.0161 (3)	0.54454 (9)	0.3420 (3)	0.0350 (7)	
C6	0.1609 (3)	0.60738 (10)	0.2772 (3)	0.0366 (7)	
C7	0.1472 (4)	0.65330 (10)	0.2934 (3)	0.0430 (7)	
H7	0.110794	0.664279	0.367734	0.052*	
C8	0.1862 (4)	0.68310 (11)	0.2016 (3)	0.0489 (8)	
H8	0.176887	0.713877	0.214109	0.059*	
C9	0.2390 (4)	0.66693 (11)	0.0915 (3)	0.0524 (9)	
H9	0.264640	0.686670	0.028400	0.063*	
C10	0.2537 (4)	0.62100 (11)	0.0749 (3)	0.0467 (8)	
H10	0.290372	0.610411	0.000239	0.056*	
C11	0.2157 (3)	0.59022 (10)	0.1660 (3)	0.0379 (7)	
C12	0.1630 (3)	0.51169 (10)	0.1725 (3)	0.0374 (7)	
C13	0.1896 (4)	0.42917 (9)	0.2035 (3)	0.0395 (7)	
H13A	0.108954	0.432885	0.262627	0.047*	
H13B	0.290178	0.424550	0.251401	0.047*	
C14	0.1511 (4)	0.38905 (10)	0.1209 (3)	0.0446 (8)	
H14A	0.149251	0.362254	0.172674	0.054*	
H14B	0.047123	0.392826	0.077708	0.054*	
C15	0.2881 (4)	0.42556 (11)	−0.0459 (3)	0.0491 (8)	
H15A	0.192261	0.430932	−0.099538	0.059*	
H15B	0.374372	0.422019	−0.099854	0.059*	
C16	0.3204 (4)	0.46547 (10)	0.0388 (3)	0.0440 (8)	
H16A	0.422192	0.461909	0.085780	0.053*	
H16B	0.323924	0.492549	−0.011672	0.053*	
C17	0.2321 (5)	0.34478 (12)	−0.0581 (4)	0.0658 (11)	
H17A	0.130021	0.349145	−0.101715	0.099*	
H17B	0.231500	0.317686	−0.009157	0.099*	
H17C	0.310467	0.342428	−0.118316	0.099*	
N1	0.2492 (3)	0.54488 (8)	0.1415 (2)	0.0422 (6)	
N2	0.1266 (3)	0.57751 (9)	0.3767 (2)	0.0401 (6)	
H2	0.104 (3)	0.5922 (10)	0.443 (3)	0.038 (9)*	
N3	0.1973 (3)	0.46954 (8)	0.1257 (2)	0.0401 (6)	
N4	0.2699 (3)	0.38349 (9)	0.0266 (2)	0.0426 (6)	
H4	0.359 (4)	0.3785 (12)	0.075 (3)	0.062 (11)*	
S1	−0.15686 (9)	0.53903 (3)	0.41676 (8)	0.0417 (2)	
C18	0.5648 (4)	0.32953 (11)	0.1890 (3)	0.0392 (7)	
C19	0.7193 (3)	0.31664 (9)	0.2566 (3)	0.0323 (6)	
C20	0.8224 (3)	0.34904 (9)	0.3104 (3)	0.0333 (6)	
H20	0.795966	0.379278	0.302211	0.040*	
C21	0.9623 (3)	0.33711 (9)	0.3753 (3)	0.0332 (6)	
C22	1.0032 (4)	0.29233 (10)	0.3851 (3)	0.0431 (8)	
H22	1.098573	0.284064	0.427658	0.052*	
C23	0.9038 (4)	0.25982 (10)	0.3322 (3)	0.0466 (8)	
H23	0.932732	0.229732	0.338820	0.056*	
C24	0.7613 (3)	0.27150 (10)	0.2692 (3)	0.0380 (7)	
O1	0.5341 (2)	0.37036 (7)	0.1732 (2)	0.0469 (6)	
O2	0.4736 (3)	0.29822 (8)	0.1504 (2)	0.0603 (7)	
O3	0.6639 (3)	0.23843 (8)	0.2203 (3)	0.0571 (7)	
H3A	0.581 (5)	0.2537 (13)	0.186 (4)	0.071 (13)*	
O4	1.0557 (3)	0.37078 (7)	0.4286 (2)	0.0442 (5)	
H4A	1.127 (5)	0.3588 (13)	0.478 (4)	0.071 (13)*	
C25	0.4829 (5)	0.37490 (14)	0.5193 (4)	0.0740 (12)	
H25A	0.463666	0.400651	0.570276	0.111*	
H25B	0.421761	0.377329	0.439808	0.111*	
H25C	0.593185	0.373497	0.505130	0.111*	
C26	0.4367 (4)	0.33350 (12)	0.5852 (3)	0.0519 (9)	
H26	0.493746	0.332991	0.668868	0.062*	
C27	0.4755 (6)	0.29132 (15)	0.5177 (4)	0.0808 (13)	
H27A	0.426807	0.292136	0.433192	0.121*	
H27B	0.436502	0.265885	0.561363	0.121*	
H27C	0.587968	0.288851	0.515149	0.121*	
O5	0.2703 (3)	0.33712 (9)	0.6017 (2)	0.0548 (6)	
H5	0.238 (4)	0.3130 (12)	0.641 (3)	0.058 (11)*	

### Atomic displacement parameters (Å^2^)

**Table:**

	*U*^11^	*U*^22^	*U*^33^	*U*^12^	*U*^13^	*U*^23^
C1	0.046 (2)	0.052 (2)	0.078 (3)	−0.0084 (16)	0.0086 (18)	−0.0055 (19)
C2	0.0369 (17)	0.0321 (16)	0.0523 (19)	0.0018 (13)	−0.0002 (14)	0.0041 (14)
C3	0.0413 (17)	0.0286 (15)	0.0445 (17)	0.0018 (13)	−0.0032 (14)	−0.0019 (13)
C4	0.0381 (17)	0.0292 (15)	0.0377 (16)	0.0041 (12)	−0.0004 (13)	0.0056 (13)
C5	0.0369 (16)	0.0324 (16)	0.0353 (16)	0.0010 (13)	0.0001 (13)	0.0057 (13)
C6	0.0335 (16)	0.0379 (17)	0.0378 (16)	−0.0064 (13)	−0.0003 (13)	0.0048 (13)
C7	0.0422 (18)	0.0393 (18)	0.0476 (18)	−0.0023 (14)	0.0044 (14)	−0.0037 (15)
C8	0.0488 (19)	0.0377 (18)	0.061 (2)	0.0000 (15)	0.0075 (16)	0.0042 (16)
C9	0.058 (2)	0.0422 (19)	0.058 (2)	−0.0043 (16)	0.0106 (17)	0.0165 (16)
C10	0.051 (2)	0.047 (2)	0.0431 (18)	−0.0029 (15)	0.0104 (15)	0.0032 (15)
C11	0.0324 (16)	0.0384 (17)	0.0429 (17)	−0.0023 (13)	0.0021 (13)	0.0042 (14)
C12	0.0416 (17)	0.0363 (17)	0.0336 (16)	0.0058 (14)	−0.0014 (13)	0.0027 (13)
C13	0.0405 (17)	0.0361 (17)	0.0424 (17)	0.0082 (13)	0.0064 (14)	0.0051 (14)
C14	0.0391 (17)	0.0388 (17)	0.055 (2)	0.0058 (14)	0.0006 (15)	−0.0009 (15)
C15	0.057 (2)	0.054 (2)	0.0368 (17)	0.0186 (17)	0.0026 (15)	0.0040 (15)
C16	0.0481 (19)	0.0451 (19)	0.0397 (17)	0.0075 (15)	0.0088 (14)	0.0053 (14)
C17	0.077 (3)	0.057 (2)	0.060 (2)	0.012 (2)	−0.017 (2)	−0.0209 (19)
N1	0.0428 (15)	0.0379 (15)	0.0469 (15)	0.0026 (12)	0.0090 (12)	0.0031 (12)
N2	0.0490 (16)	0.0389 (15)	0.0321 (14)	−0.0091 (12)	0.0011 (12)	−0.0037 (12)
N3	0.0484 (15)	0.0334 (14)	0.0396 (14)	0.0055 (11)	0.0096 (12)	0.0037 (11)
N4	0.0431 (15)	0.0422 (15)	0.0408 (15)	0.0124 (12)	−0.0072 (13)	−0.0056 (12)
S1	0.0447 (5)	0.0361 (4)	0.0455 (5)	−0.0030 (3)	0.0105 (4)	−0.0033 (3)
C18	0.0372 (17)	0.0459 (19)	0.0346 (16)	0.0043 (15)	0.0032 (13)	−0.0043 (14)
C19	0.0315 (15)	0.0368 (16)	0.0294 (14)	0.0039 (12)	0.0063 (12)	−0.0023 (12)
C20	0.0379 (16)	0.0268 (14)	0.0354 (15)	0.0050 (12)	0.0047 (13)	0.0017 (12)
C21	0.0363 (16)	0.0327 (16)	0.0307 (14)	−0.0023 (12)	0.0028 (12)	0.0000 (12)
C22	0.0397 (17)	0.0405 (18)	0.0472 (18)	0.0078 (14)	−0.0085 (14)	0.0035 (14)
C23	0.0491 (19)	0.0253 (16)	0.064 (2)	0.0052 (14)	−0.0056 (16)	−0.0023 (15)
C24	0.0359 (16)	0.0348 (16)	0.0430 (17)	−0.0012 (13)	0.0022 (13)	−0.0060 (13)
O1	0.0411 (12)	0.0462 (14)	0.0518 (13)	0.0106 (10)	−0.0059 (10)	0.0003 (10)
O2	0.0412 (13)	0.0572 (15)	0.0789 (18)	−0.0010 (12)	−0.0182 (12)	−0.0084 (13)
O3	0.0453 (14)	0.0362 (13)	0.0874 (19)	−0.0014 (11)	−0.0101 (13)	−0.0156 (12)
O4	0.0440 (13)	0.0357 (12)	0.0507 (13)	−0.0039 (10)	−0.0116 (11)	0.0016 (10)
C25	0.051 (2)	0.083 (3)	0.089 (3)	−0.008 (2)	0.006 (2)	0.022 (2)
C26	0.0376 (18)	0.065 (2)	0.052 (2)	−0.0007 (16)	−0.0038 (15)	0.0092 (17)
C27	0.087 (3)	0.077 (3)	0.083 (3)	−0.001 (2)	0.034 (3)	−0.006 (2)
O5	0.0398 (13)	0.0617 (16)	0.0624 (16)	−0.0048 (11)	−0.0001 (11)	0.0184 (13)

### Geometric parameters (Å, º)

**Table:**

C1—C2	1.496 (4)	C16—N3	1.452 (4)
C1—H1A	0.9600	C16—H16A	0.9700
C1—H1B	0.9600	C16—H16B	0.9700
C1—H1C	0.9600	C17—N4	1.483 (4)
C2—C3	1.343 (4)	C17—H17A	0.9600
C2—S1	1.736 (3)	C17—H17B	0.9600
C3—C4	1.434 (4)	C17—H17C	0.9600
C3—H3	0.9300	N2—H2	0.87 (3)
C4—C5	1.365 (4)	N4—H4	0.89 (4)
C4—C12	1.474 (4)	C18—O1	1.252 (4)
C5—N2	1.388 (4)	C18—O2	1.260 (4)
C5—S1	1.729 (3)	C18—C19	1.496 (4)
C6—C7	1.383 (4)	C19—C24	1.394 (4)
C6—C11	1.400 (4)	C19—C20	1.395 (4)
C6—N2	1.429 (4)	C20—C21	1.373 (4)
C7—C8	1.378 (4)	C20—H20	0.9300
C7—H7	0.9300	C21—O4	1.373 (3)
C8—C9	1.372 (5)	C21—C22	1.379 (4)
C8—H8	0.9300	C22—C23	1.376 (4)
C9—C10	1.385 (4)	C22—H22	0.9300
C9—H9	0.9300	C23—C24	1.381 (4)
C10—C11	1.388 (4)	C23—H23	0.9300
C10—H10	0.9300	C24—O3	1.363 (4)
C11—N1	1.408 (4)	O3—H3A	0.89 (4)
C12—N1	1.287 (4)	O4—H4A	0.85 (4)
C12—N3	1.389 (4)	C25—C26	1.485 (5)
C13—N3	1.463 (4)	C25—H25A	0.9600
C13—C14	1.503 (4)	C25—H25B	0.9600
C13—H13A	0.9700	C25—H25C	0.9600
C13—H13B	0.9700	C26—O5	1.440 (4)
C14—N4	1.484 (4)	C26—C27	1.495 (5)
C14—H14A	0.9700	C26—H26	0.9800
C14—H14B	0.9700	C27—H27A	0.9600
C15—N4	1.485 (4)	C27—H27B	0.9600
C15—C16	1.503 (4)	C27—H27C	0.9600
C15—H15A	0.9700	O5—H5	0.88 (4)
C15—H15B	0.9700		
			
C2—C1—H1A	109.5	N4—C17—H17A	109.5
C2—C1—H1B	109.5	N4—C17—H17B	109.5
H1A—C1—H1B	109.5	H17A—C17—H17B	109.5
C2—C1—H1C	109.5	N4—C17—H17C	109.5
H1A—C1—H1C	109.5	H17A—C17—H17C	109.5
H1B—C1—H1C	109.5	H17B—C17—H17C	109.5
C3—C2—C1	130.5 (3)	C12—N1—C11	124.2 (3)
C3—C2—S1	110.5 (2)	C5—N2—C6	114.5 (2)
C1—C2—S1	119.1 (2)	C5—N2—H2	113 (2)
C2—C3—C4	114.5 (3)	C6—N2—H2	111 (2)
C2—C3—H3	122.7	C12—N3—C16	119.0 (2)
C4—C3—H3	122.7	C12—N3—C13	121.3 (2)
C5—C4—C3	111.5 (3)	C16—N3—C13	110.9 (2)
C5—C4—C12	120.9 (3)	C17—N4—C14	111.8 (3)
C3—C4—C12	127.6 (3)	C17—N4—C15	111.5 (3)
C4—C5—N2	126.8 (3)	C14—N4—C15	111.1 (2)
C4—C5—S1	111.5 (2)	C17—N4—H4	111 (2)
N2—C5—S1	121.7 (2)	C14—N4—H4	103 (2)
C7—C6—C11	120.1 (3)	C15—N4—H4	109 (2)
C7—C6—N2	119.9 (3)	C5—S1—C2	91.97 (14)
C11—C6—N2	119.9 (3)	O1—C18—O2	123.9 (3)
C8—C7—C6	121.4 (3)	O1—C18—C19	118.6 (3)
C8—C7—H7	119.3	O2—C18—C19	117.5 (3)
C6—C7—H7	119.3	C24—C19—C20	118.6 (3)
C9—C8—C7	119.4 (3)	C24—C19—C18	120.1 (3)
C9—C8—H8	120.3	C20—C19—C18	121.3 (3)
C7—C8—H8	120.3	C21—C20—C19	121.2 (3)
C8—C9—C10	119.5 (3)	C21—C20—H20	119.4
C8—C9—H9	120.2	C19—C20—H20	119.4
C10—C9—H9	120.2	O4—C21—C20	117.9 (3)
C9—C10—C11	122.3 (3)	O4—C21—C22	122.7 (3)
C9—C10—H10	118.8	C20—C21—C22	119.4 (3)
C11—C10—H10	118.8	C23—C22—C21	120.4 (3)
C10—C11—C6	117.3 (3)	C23—C22—H22	119.8
C10—C11—N1	116.3 (3)	C21—C22—H22	119.8
C6—C11—N1	126.2 (3)	C22—C23—C24	120.5 (3)
N1—C12—N3	117.6 (3)	C22—C23—H23	119.7
N1—C12—C4	126.6 (3)	C24—C23—H23	119.7
N3—C12—C4	115.7 (3)	O3—C24—C23	119.1 (3)
N3—C13—C14	109.8 (2)	O3—C24—C19	121.1 (3)
N3—C13—H13A	109.7	C23—C24—C19	119.8 (3)
C14—C13—H13A	109.7	C24—O3—H3A	103 (2)
N3—C13—H13B	109.7	C21—O4—H4A	108 (3)
C14—C13—H13B	109.7	C26—C25—H25A	109.5
H13A—C13—H13B	108.2	C26—C25—H25B	109.5
N4—C14—C13	110.8 (2)	H25A—C25—H25B	109.5
N4—C14—H14A	109.5	C26—C25—H25C	109.5
C13—C14—H14A	109.5	H25A—C25—H25C	109.5
N4—C14—H14B	109.5	H25B—C25—H25C	109.5
C13—C14—H14B	109.5	O5—C26—C25	107.0 (3)
H14A—C14—H14B	108.1	O5—C26—C27	112.0 (3)
N4—C15—C16	112.1 (3)	C25—C26—C27	113.2 (3)
N4—C15—H15A	109.2	O5—C26—H26	108.1
C16—C15—H15A	109.2	C25—C26—H26	108.1
N4—C15—H15B	109.2	C27—C26—H26	108.1
C16—C15—H15B	109.2	C26—C27—H27A	109.5
H15A—C15—H15B	107.9	C26—C27—H27B	109.5
N3—C16—C15	109.9 (3)	H27A—C27—H27B	109.5
N3—C16—H16A	109.7	C26—C27—H27C	109.5
C15—C16—H16A	109.7	H27A—C27—H27C	109.5
N3—C16—H16B	109.7	H27B—C27—H27C	109.5
C15—C16—H16B	109.7	C26—O5—H5	110 (2)
H16A—C16—H16B	108.2		
			
C1—C2—C3—C4	−179.3 (3)	N1—C12—N3—C16	−3.9 (4)
S1—C2—C3—C4	0.6 (3)	C4—C12—N3—C16	172.5 (3)
C2—C3—C4—C5	−0.6 (4)	N1—C12—N3—C13	140.6 (3)
C2—C3—C4—C12	177.8 (3)	C4—C12—N3—C13	−42.9 (4)
C3—C4—C5—N2	−178.0 (3)	C15—C16—N3—C12	−152.5 (3)
C12—C4—C5—N2	3.5 (4)	C15—C16—N3—C13	59.5 (3)
C3—C4—C5—S1	0.3 (3)	C14—C13—N3—C12	151.8 (3)
C12—C4—C5—S1	−178.3 (2)	C14—C13—N3—C16	−61.1 (3)
C11—C6—C7—C8	0.1 (5)	C13—C14—N4—C17	−178.6 (3)
N2—C6—C7—C8	177.0 (3)	C13—C14—N4—C15	−53.4 (3)
C6—C7—C8—C9	0.4 (5)	C16—C15—N4—C17	177.9 (3)
C7—C8—C9—C10	−0.7 (5)	C16—C15—N4—C14	52.5 (4)
C8—C9—C10—C11	0.5 (5)	C4—C5—S1—C2	0.1 (2)
C9—C10—C11—C6	0.0 (5)	N2—C5—S1—C2	178.4 (2)
C9—C10—C11—N1	−174.7 (3)	C3—C2—S1—C5	−0.4 (2)
C7—C6—C11—C10	−0.3 (4)	C1—C2—S1—C5	179.6 (3)
N2—C6—C11—C10	−177.2 (3)	O1—C18—C19—C24	−176.1 (3)
C7—C6—C11—N1	173.8 (3)	O2—C18—C19—C24	3.3 (4)
N2—C6—C11—N1	−3.1 (5)	O1—C18—C19—C20	5.2 (4)
C5—C4—C12—N1	−34.3 (4)	O2—C18—C19—C20	−175.4 (3)
C3—C4—C12—N1	147.5 (3)	C24—C19—C20—C21	−0.4 (4)
C5—C4—C12—N3	149.7 (3)	C18—C19—C20—C21	178.3 (3)
C3—C4—C12—N3	−28.6 (4)	C19—C20—C21—O4	−178.3 (2)
N3—C13—C14—N4	57.4 (3)	C19—C20—C21—C22	1.5 (4)
N4—C15—C16—N3	−55.2 (3)	O4—C21—C22—C23	178.6 (3)
N3—C12—N1—C11	170.5 (3)	C20—C21—C22—C23	−1.1 (5)
C4—C12—N1—C11	−5.5 (5)	C21—C22—C23—C24	−0.4 (5)
C10—C11—N1—C12	−144.3 (3)	C22—C23—C24—O3	−178.5 (3)
C6—C11—N1—C12	41.5 (5)	C22—C23—C24—C19	1.5 (5)
C4—C5—N2—C6	56.1 (4)	C20—C19—C24—O3	178.9 (3)
S1—C5—N2—C6	−121.9 (3)	C18—C19—C24—O3	0.2 (4)
C7—C6—N2—C5	125.9 (3)	C20—C19—C24—C23	−1.1 (4)
C11—C6—N2—C5	−57.2 (4)	C18—C19—C24—C23	−179.7 (3)

### Hydrogen-bond geometry (Å, º)

**Table:**

*D*—H···*A*	*D*—H	H···*A*	*D*···*A*	*D*—H···*A*
C1—H1*A*···O1^i^	0.96	2.64	3.579 (4)	168
C10—H10···O1^ii^	0.93	2.52	3.316 (4)	143
C13—H13*A*···O4^i^	0.97	2.62	3.233 (4)	122
C17—H17*B*···O2	0.96	2.63	3.216 (4)	120
N2—H2···O4^iii^	0.87 (3)	2.28 (3)	3.088 (4)	156 (3)
N4—H4···O1	0.89 (4)	1.77 (4)	2.660 (3)	178 (4)
O3—H3*A*···O2	0.89 (4)	1.63 (4)	2.479 (3)	156 (4)
O4—H4*A*···O5^iv^	0.85 (4)	1.84 (4)	2.682 (3)	173 (4)
O5—H5···O3^v^	0.88 (4)	1.88 (4)	2.764 (3)	178 (3)

Symmetry codes: (i) *x*−1, *y*, *z*; (ii) −*x*+1, −*y*+1, −*z*; (iii) −*x*+1, −*y*+1, −*z*+1; (iv) *x*+1, *y*, *z*; (v) *x*−1/2, −*y*+1/2, *z*+1/2.
